# Outcomes in children with *Clostridium difficile* infection: results from a nationwide survey

**DOI:** 10.1093/gastro/gow007

**Published:** 2016-04-14

**Authors:** Arjun Gupta, Darrell S Pardi, Larry M Baddour, Sahil Khanna

**Affiliations:** ^1^Divisions of Infectious Diseases, Mayo Clinic, Rochester, MN, USA; ^2^Divisions of Gastroenterology and Hepatology, Mayo Clinic, Rochester, MN, USA; ^3^Department of Internal Medicine, University of Texas Southwestern Medical Center, Dallas, TX, USA

**Keywords:** *Clostridium difficile* infection, children, pediatric, outcomes, epidemiology

## Abstract

**Objective:** Hospital- and population-based studies demonstrate an increasing incidence of *Clostridium difficile* infection (CDI) in adults and children; although pediatric CDI outcomes are incompletely understood. We analysed United States National Hospital Discharge Survey (NHDS) data to study CDI in hospitalized children.

**Methods:** NHDS data for 2005–2009 (demographics, diagnoses and discharge status) were obtained; cases and comorbidities were identified using ICD-9 codes. Weighted univariate and multivariate analyses were performed to ascertain incidence of CDI; associations between CDI and outcomes [length of stay (LOS), colectomy, all-cause in-hospital mortality and discharge to a care facility (DTCF)].

**Results:** Of an estimated 13.8 million pediatric inpatients; 46 176 had CDI; median age was 3 years; overall incidence was 33.5/10 000 hospitalizations. The annual frequency of CDI did not vary from 2005 to 2009 (0.24–0.43%; *P* = 0.64). On univariate analyses, children with CDI had a longer median LOS (6 *vs* 2 days), higher rates of colectomy [odds ratio (OR) 2.0; 95% confidence interval (CI) 1.7–2.4], mortality (OR 2.5; 95% CI 2.3–2.7), and DTCF (OR 1.6; 95% CI 1.6–1.7) (all *P* < 0.0001). After adjusting for age, sex and comorbidities, CDI was an independent and the strongest predictor of increased LOS (adjusted mean difference, 6.4 days; 95% CI 5.4–7.4), higher rates of colectomy (OR 2.1; 95% CI 1.8–2.5), mortality (OR 2.3; 95% CI 2.2–2.5), and DTCF (OR 1.7; 95% CI 1.6–1.8) (all *P* < 0.0001). On excluding infants from the analysis, children with CDI had higher rates of mortality, DTCF and longer LOS than children without CDI.

**Conclusions**: Despite increased awareness and advancements in management, CDI remains a significant problem and is associated with increased LOS, colectomy, in-hospital mortality and DTCF in hospitalized children.

## Introduction

*Clostridium difficile* is the most common healthcare-associated infection [1] and the principal cause of infectious diarrhea in hospitalized patients [2]. *C. difficile* infection (CDI) is associated with known risk factors, including hospitalization, advanced age, gastrointestinal surgery or procedures, and antibiotic exposure [2]. The disease spectrum of CDI ranges from mild to severe colitis and can be complicated by recurrent infection, sepsis, need for critical care, surgery or death. CDI has also emerged in populations previously considered to be at low risk and lacking the traditional risk factors for CDI [3], including in the community setting [4]. Recent studies have shown that CDI is a more common cause of infectious diarrhea in children than previously thought, both in the hospital and community settings, with growing incidence and severity [5–9]. Outbreaks of pediatric CDI have also been reported [10, 11]. An analysis of National Hospital Discharge Survey (NHDS) data from the USA showed an increasing incidence of CDI in hospitalized children from 1997 to 2006 [12]; however, there is limited information on outcomes in respect of CDI in children, including the effect of CDI on length of hospital stay, in-hospital mortality, colectomy and discharge to a care facility. In the current study, we analysed United States NHDS data from 2005–2009 to evaluate these outcomes in pediatric patients with CDI.

## Materials and methods

### Data source

The National Hospital Discharge Survey (NHDS) has been conducted annually in the USA since 1965 and collects hospital discharge information from non-federal short-stay hospitals [defined as average length of stay (LOS) less than 30 days] throughout the United States with a stratified random sampling process. NHDS contains diagnosis and procedure codes, demographics, admission type, LOS, all-cause in-hospital mortality, and discharge information (e.g. to home or to a short-term or long-term healthcare facility). The database is publicly available online at http://www.cdc.gov/nchs/nhds.htm. Diagnoses are based on the International Classification of Diseases, Ninth Revision, Clinical Modification (ICD-9-CM) codes.

### Data collection

Data extraction and statistical analysis were carried out using Statistical Analysis Software (SAS) version 9.2 and JMP version 9.01 (SAS Institute, Cary, NC, USA). Data collected and analysed for this study included age, sex, race, admission type (urgent or emergent versus elective), any diagnosis of colectomy, length of stay, type of discharge, and mortality for all patients discharged between January 1, 2005, through December 31, 2009.

### Definition of variables

Patients recorded in the NHDS database from 2005–2009 with age <18 years, with an ICD-9-CM code of 008.45 listed as their primary or additional diagnosis (from 2005–2009) or diagnosis on admission (data collected 2008 onwards), were deemed to be CDI patients. Individuals born during that hospital admission (coded as newborns) were excluded. We analysed outcomes in children aged 2–17 years separately after excluding infants (aged <1 year).

#### Race

Data on race collected by NHDS is classified into White, Black / African American, American Indian / Alaskan Native, Asian, Native Hawaiian / Other Pacific Islander, Other, Multiple race indicated, or unknown.

#### Geographical region

Hospitalizations were classified according to geographical area of the United States: Northeast, Midwest, South and West. Hawaii and Alaska were included in the ‘West’ geographical area.

#### Admission type

Hospital admissions in pediatric patients in the NHDS database are characterized as emergency, urgent, elective or not available.

#### Discharge type

The type of hospital discharge is classified by the NHDS database into the following categories: routine and/or discharged home, discharged to a short-term healthcare facility, discharged to a long-term healthcare facility, unknown discharge status, left against medical advice, or death during hospitalization. To analyse the likelihood of discharge to a care-term facility (DTCF), we combined all patients discharged to short- and long-term healthcare facilities and compared them with patients who had a routine and/or home discharge. Patients who died or for whom the type of discharge was not stated were excluded from this aspect of the analysis.

#### Surgical procedures

ICD-9 codes that were used to determine which patients underwent partial or total colectomy included 45.71, 45.72, 45.73, 45.74, 45.75 and 45.76, 45.8, 45.81, 45.82 and 45.83. The incidence of colectomy was compared in patients with and without CDI.

#### Length of stay

The NHDS collects data on the LOS for all patients. These data were abstracted electronically and used to calculate differences in LOS among patients with CDI, as compared with other patients.

#### In-hospital mortality

Death during hospitalization was analysed as a separate clinical outcome. Mortality after release from hospital is not included in the data collected by the NHDS. All-cause mortality for CDI was compared with that for pediatric patients without CDI.

#### Comorbidities

Comorbid conditions were abstracted from the data using the Health-care cost and utilization guidelines at https://www.hcup-us.ahrq.gov/toolssoftware/comorbidity/comorbidity.jsp. Clinically relevant comorbid conditions including hematological malignancies, solid organ tumors, inflammatory bowel disease and chronic lung disease were abstracted and used for statistical adjustment along with age and sex. Other comorbidities were not used due to the low frequency of those conditions in this patient population.

### Statistical analyses

The summary database was converted to a JMP file (SAS Institute, Cary, NC, USA) using SAS version 9.2. Demographic and clinical outcome data were analysed using the *t*-test for normally distributed continuous variables and the Wilcoxon rank sum test if not normally distributed (e.g. age and LOS). For comparison of continuous data among several groups, analysis of variance was used if the data were normally distributed, and the Kruskal-Wallis test was used if data were skewed. Results of continuous variables are reported as mean or median (minimum, maximum) as appropriate. Categorical variables are reported as percentages and compared using odds ratios (ORs) and 95% confidence intervals (95% CIs). Multivariate linear and logistic regression models with weighted analyses were used to adjust for the effect of age, sex and comorbid conditions on CDI-associated outcomes. Weighted analysis was performed in order to obtain nationwide estimates and account for the stratified sampling process of the NHDS database. A *P*-value of less than 0.01 was considered statistically significant.

## Results

### Patient characteristics

From 2005–2009, the NHDS database included an estimated 13.8 million pediatric hospital admissions. These patients had a median age of 5 years (range 1–17) and 47.8% were female. Overall, 72.4% admissions were classified as emergency or urgent. The median LOS was 2 days (range 1–561) and 2.7% of all patients were dismissed to a short- or long-term care facility. The overall rate of colectomy was 0.2% and the overall all-cause in-hospital mortality was 0.5%. Detailed patient characteristics are shown in Table 1.

**Table 1.** Patient characteristics and incidence of *Clostridium difficile* infection

Characteristics	Entire cohort (*n = * 13.8 x 10^6)^	Patients with CDI (*n = * 46, 176)	Patients without CDI (*n = * 13.7 x 10^6^)	Incidence of CDI/ 10 000 hospitalizations	*P*-value^a^
Median age, years	5 (2–14)	3 (2–11)	5 (2–14)		
Age distribution					
<1 year	26.3	27.8	26.3	35.4	<0.0001
1–17 years	73.7	72.2	73.7	32.8	
Race					
Caucasian	56.7	52.7	56.7	31.5	<0.0001
African-American	14.7	12.1	14.7	27.7	
Asian	1.4	2.1	1.4	50.8	
Others^b^	27.2	33.1	27.2	40.6	
Geographical region					
Northeast	18.5	20.4	18.5	36.9	<0.0001
Midwest	15.5	13.0	15.5	28.1	
South	38.6	30.9	38.6	26.8	
West	27.4	35.7	27.4	43.7	
Admission type					
Emergency	41.7	9.1	41.7	31.4	<0.0001
Urgent	30.7	30.6	30.7	33.4	
Elective	19.4	23.2	19.4	40.0	
Unknown	8.2	7.1	8.2	28.9	
Hospital ownership					
Proprietary	10.4	7.5	10.4	24.2	<0.0001
Government	11.3	7.9	11.3	23.4	
Non-profit	78.3	84.6	78.3	36.2	
Hospital beds					
6–99	15.2	7.9	15.2	17.5	<0.0001
100–199	18.1	17.6	18.1	32.6	
200–299	30.3	33.4	30.3	36.9	
300–499	22.8	24.6	22.8	36.1	
≥500	13.6	16.5	13.6	40.7	

All data are proportion of cases unless otherwise stated.

^a^*P*-value for comparing incidence of CDI in the various groups.

^b^‘Others’ includes patients in whom the race was not stated or multiple races were indicated for the same patient.

CDI = *C. difficile* infection

**Figure 1.**
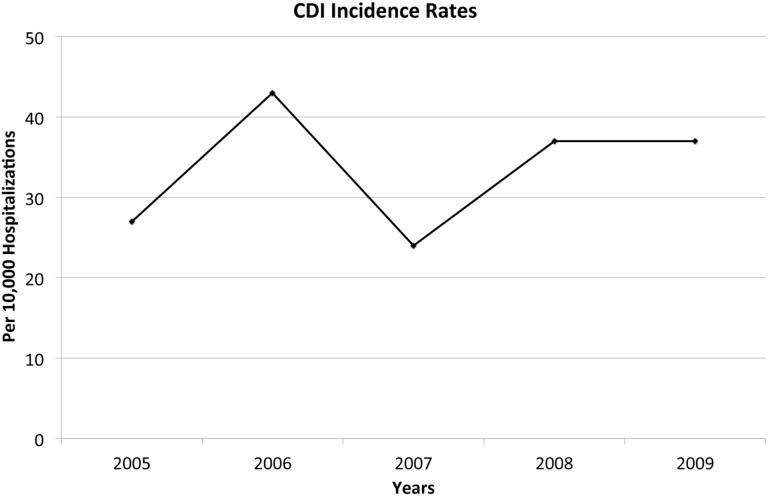
Annual rate of incidence of *Clostridium difficile* infection over the study period.

There were 46 176 pediatric CDI cases with an overall incidence of 33.5/10 000 hospitalizations. The annual incidence of CDI varied from 24 to 43 cases per 10 000 pediatric hospitalizations over the study period, with no significant trend (*P* = 0.64) (Figure 1). Also no significant trend was seen over the study period in the rates of colectomy, in-hospital mortality, DTCF and LOS in pediatric CDI cases. Among geographical regions, the Western region had the highest incidence of hospitalizations with CDI, followed by the Northeast. There was a significantly higher incidence of CDI in hospitals with larger numbers of beds signifying that larger hospitals have a higher CDI incidence. (Table 1).

Of the estimated 13.8 million pediatric inpatients, 3.6 million infants accounted for 26% of the population. The rates of CDI in infants and in those aged 2–17 years were similar (35 *vs* 33 per 10 000 hospitalizations, respectively).

### Outcomes from CDI

#### Length of stay

The median LOS among all hospitalized children was 2.0 days. Patients with CDI had a median LOS of 6.0 days. After adjusting for co-morbid conditions, CDI was the strongest independent predictor of increased LOS among predictors analysed (adjusted mean difference in LOS 6.4 days; 95% CI 5.4–7.4; *P* < 0.0001) (Table 2).

**Table 2.** Comparison of outcomes in patients with and without CDI

Outcomes	Patients with CDI	Patients without CDI	OR (95% CI)	Adjusted OR (95% CI)	*P*-value^a^
Median length of stay (days)	6	2	Mean difference: 4	Adjusted mean difference: 6.4 (5.4–7.4)	<0.0001
In-hospital mortality, %	1.2	0.5	2.5 (2.3–2.7)	2.3 (2.2–2.5)	<0.0001
Colectomy, %	0.3	0.2	2.1 (1.7–2.4)	2.1 (1.8–2.5)	<0.0001
Discharge to care facility, % ^b^	4.3	2.7	1.6 (1.6–1.7)	1.7 (1.6–1.8)	<0.0001

Data are presented as number (percentage) or median (minimum, maximum).

^a^*P*-value for the adjusted OR. Adjustment was made for age, sex, and comorbidities, including hematological malignancies, solid tumors and chronic lung disease.

^b^Excluding patients who left against medical advice, died or in whom discharge status was not reported.

CDI = *C. difficil*e infection; CI = confidence interval; OR = odds ratio.

#### Colectomy

The overall rate of colectomy in all hospitalized patients was 0.2%. Patients with CDI showed a higher likelihood of undergoing colectomy than those without CDI. After adjusting for co-morbidities, CDI was an independent predictor of colectomy in these patients (Table 2).

#### All-cause in-hospital mortality

The overall in-hospital mortality was 0.5%. Patients with CDI had a higher all-cause mortality than patients without the disease. After adjusting for co-morbidities, CDI was an independent predictor of all-cause in-hospital mortality (Table 2).

#### Hospital discharge

The overall rate of discharge to a short- or long-term care facility was 2.7%. Patients with CDI were more likely to be discharged to a care facility than those without CDI. After adjusting for co-morbidities, CDI was an independent predictor of DTCF (Table 2).

#### *Outcomes in children aged 2*–*17 years (excluding infants)*

On analysing data restricting the study population to non-infants (age 2–17 years), the rates of DTCF and mortality, and the mean LOS, remained significantly higher in the CDI group than in *the* non-CDI group; however there was no significant difference in rates of colectomy between children with or without CDI (Table 3).

**Table 3.** Comparison of outcomes in children aged 2–17 years with and without CDI

Outcomes	Patients with CDI	Patients without CDI	OR (95% CI)	Adjusted OR (95% CI)	*P*-value^a^
Median length of stay (days)	6	2	Mean difference: 4	Adjusted mean difference: 5.8 (4.9–6.5)	<0.0001
In-hospital mortality, %	1.7	0.3	5.9 (5.4–6.4)	6.2 (5.7–6.7)	<0.0001
Colectomy, %^b^	0.1	0.1	0.9 (0.6–1.2)	1.0 (0.7–1.3)	–
Discharge to care facility, %^c^	4.0	2.5	1.7 (1.6–1.8)	1.6 (1.6–1.7)	<0.0001

Data are presented as number (percentage) or median (minimum, maximum).

^a^*P*-value for the adjusted OR. Adjustment was made for age, sex, and co-morbidities, including hematological malignancies, solid tumors, chronic lung disease and inflammatory bowel disease.

^b^No adjustment for IBD made as no IBD and CDI cases had colectomy.

^c^Excluding patients who left against medical advice, died or in whom discharge status was not reported.

CDI = *C. difficile* infection; CI = confidence interval; OR = odds ratio.

#### Relationship to inflammatory bowel disease

Of the estimated 13.8 million pediatric inpatients, an estimated 31 842 had a diagnosis of *inflammatory bowel disease* (IBD). This equates to a rate of 23 cases per 10 000 hospitalizations. The rates of CDI were much higher in children with IBD (415/10 000 hospitalizations) than in those without (33/10 000 hospitalizations). Rates of colectomy were significantly higher in IBD patients without CDI (11.5%) than in those with CDI (0%).

## Discussion

We have demonstrated, using a large national database, that CDI is associated with worse outcomes in hospitalized children including longer LOS, higher rates of colectomy, higher likelihood of DTCF and higher in-hospital mortality. Even after adjusting for comorbid conditions, CDI remained the strongest independent predictor of these adverse outcomes compared to comorbid conditions in the multivariate analysis.

Numerous studies have shown rising rates of adult and pediatric CDI over the past decade [7, 13–15]. There have been reports that incidence of CDI has peaked and is now reaching a plateau, and there was no significant increase in the incidence of CDI in other large studies in children [5, 16]. Change in the incidence of CDI over time may represent a true increase due to the emergence of a hypervirulent strain, an increase in ‘at risk’ population owing to increasing use of antibiotics, more sensitive testing methods and better coding over time. An increase in incidence was not demonstrated in our study, perhaps reflecting the relatively short 5-year study period and the slowing of the CDI epidemic in the latter half of the last decade. Increased incidence of CDI in larger hospitals was noted; this could be explained by more frequent testing for *C. difficile* in larger hospitals, or the presentation of sicker children with CDI to these institutions, or lapses in infection control practices.

Whether pediatric CDI is merely an extension of adult CDI, or has its own specific epidemiology—and might actually be a source of- and magnify adult CDI—is debatable. Among the different pediatric age groups, a maximum increase in incidence has been noted in the diaper-wearing 1–4 year-old population [14]. Finding similar *C. difficile* strains in pediatric and adult patients, and increasing community-acquired CDI (CA-CDI) in adults, lend support to the hypothesis that pediatric patients can act as sources for adult CDI [17]. A case control study identified close contact with children under the age of 2 years as a potential risk factor for CA-CDI [18]. A plausible role for infants and young children acting as reservoirs and vectors for *C. diffic*ile is supported by data that several toxigenic and non-toxigenic strains are carried by infants, although none was found to carry the hypervirulent 027 or 078 strains [19, 20]. Regular diaper changing, by mothers, in babies carrying *C. difficile* has been hypothesized to partially explain the female predilection of CA-CDI [21].

The large difference between colectomy rates in IBD patients without CDI (11.5%) and with CDI (0%) is interesting and may indicate the predominantly non-surgical therapy for CDI in children, primarily in IBD patients and how sick these children with IBD and CDI may be, precluding them from getting a colectomy. The fact that children aged 2–17 years with CDI did not have higher rates of colectomy than children without CDI probably points at the rarity of colectomy as a therapeutic agent for CDI in children (10/10 000 hospitalizations in both groups).

Reports on the severity of CDI and outcomes in children have demonstrated heterogenous results. Our group has previously shown a high rate of adverse outcomes in children with CDI in a population-based study [13]. Other reports have shown low rates of CDI-associated complications [16]. An increase in complications over time has not been noted in previous longitudinal studies [7, 22]. One source of heterogeneity may be the use of non-standardized definitions for severe infection [23]; defined criteria for severity of CDI exist for adults but are lacking for children [24].

Analyses have in the past excluded infants [8]. Pediatric diarrhea has historically been considered primarily a viral disease, but a recent study reported *C. difficile* as the most common single organism isolated from the stool of children with healthcare-associated diarrhea [25]. Although originally thought to be non-pathogenic in infants [26], CDI has been shown to afflict infants, as shown by the isolation of *C. difficile* from their stools, absence of any other identified cause of diarrhea, and response to metronidazole therapy. The American Academy of Pediatrics recommends testing for CDI if the clinical suspicion is high but recommends first considering alternate diagnoses. We included all inpatients—other than newborns—in our study, identically to other studies [7], but also analysed data after excluding infants. Due to the risk of colonization, the significance of positive *C. difficile* testing in infants remains unclear, and such findings should be interpreted with caution [27]. Thirty to forty percent of adult inpatients with a positive stool assay for *C. difficile* are colonized but do not have colitis (asymptomatic carriers).

Length of hospital stay has a dual relationship with CDI as increased length of hospital stay is a well-known risk factor for CDI, and patients with CDI tend to stay longer in the hospital.

There are several limitations to our study. The data were analysed as part of a large national survey, and longitudinal follow-up for patients is not available. Being an administrative database, the NHDS lacks information on risk factors such as exposure to antibiotics or PPIs, CDI treatment, *C. difficile* strain, severity of CDI, healthcare-associated *vs* CA-CDI, and important laboratory parameters such as serum creatinine or leukocyte count. Several studies have failed to demonstrate an association between use of PPIs and CDI in children [15, 28, 29] and, although a majority of children with CDI had received antibiotics in a large population-based study from Olmsted County, MN, USA, a fifth did not receive antibiotics or PPIs, while low rates of antibiotic usage have been reported in other studies [5, 13]. There is limited information on CDI colonization *vs* true infection, as the data are based on ICD-9 codes. Nevertheless, the ICD-9-CM code for CDI diagnosis has been validated in previous studies, and has been shown to correlate well with the results of *C. difficile* toxin assay [30, 31].

## Conclusions

Despite increased awareness of CDI in children and advancements in management and infection prevention and control practices, CDI remains a problem in hospitalized children, and is an independent predictor of increased LOS and poor outcomes such as colectomy, in-hospital mortality and DTCF. Several actions are needed, including more aggressive policies on infection control, antimicrobial stewardship and education to enhance the early recognition and prompt treatment of CDI, hopefully to prevent adverse outcomes and reduce the spread of infection.

## Funding

None

*Conflict of interest statement*: none declared.

## Author contributions

AG, DSP, LMB and SK designed the research, analysed the data, wrote the paper and critically revised the manuscript for important intellectual content.

## Data sharing statement

no additional data available.

## IRB statement

IRB approval was not required as the research data is held in the public domain.

## Informed consent statement

Data were obtained from the NHDS, which is in the public domain.
